# Harnessing synthetic biology for advancing RNA therapeutics and vaccine design

**DOI:** 10.1038/s41540-023-00323-3

**Published:** 2023-11-30

**Authors:** Blaine A. Pfeifer, Marie Beitelshees, Andrew Hill, Justin Bassett, Charles H. Jones

**Affiliations:** 1 Department of Chemical and Biological Engineering, University at Buffalo, The State University of New York, Buffalo, NY USA; 2 Pfizer, 66 Hudson Boulevard, New York, NY 10001 USA

**Keywords:** Synthetic biology, Synthetic biology, Synthetic biology

## Abstract

Recent global events have drawn into focus the diversity of options for combatting disease across a spectrum of prophylactic and therapeutic approaches. The recent success of the mRNA-based COVID-19 vaccines has paved the way for RNA-based treatments to revolutionize the pharmaceutical industry. However, historical treatment options are continuously updated and reimagined in the context of novel technical developments, such as those facilitated through the application of synthetic biology. When it comes to the development of genetic forms of therapies and vaccines, synthetic biology offers diverse tools and approaches to influence the content, dosage, and breadth of treatment with the prospect of economic advantage provided in time and cost benefits. This can be achieved by utilizing the broad tools within this discipline to enhance the functionality and efficacy of pharmaceutical agent sequences. This review will describe how synthetic biology principles can augment RNA-based treatments through optimizing not only the vaccine antigen, therapeutic construct, therapeutic activity, and delivery vector. The enhancement of RNA vaccine technology through implementing synthetic biology has the potential to shape the next generation of vaccines and therapeutics.

## Introduction

The pharmaceutical industry has long depended on synthetic chemistry approaches to develop novel therapeutics. These approaches, which have led to numerous breakthroughs in drug development, have typically relied on screening compound libraries populated by a range of molecules derived from a set of known and robust chemistry reactions in order to achieve improved efficacy, safety, and pharmacokinetic properties^1^. However, they often fall short when addressing complex diseases or diseases with genetic roots. Additionally, the cost and extended development timelines associated with these approaches have the potential to limit their application to emergent diseases such as SARS-CoV-2. Synthetic biology, a field that has already had a significant impact on the agricultural, environmental, and renewable biofuel industries^2^, has emerged as an attractive alternative drug development approach.

Synthetic biology is a scientific field that involves the rewiring of organisms or biomolecular parts for new and desired abilities. This encompasses diverse applications, from engineering new or improved activities in enzymes, heterologous production of commodity chemicals, assembly of genetic parts in a synthetic manner and developing cellular therapies. Although synthetic biology itself is a relatively new field that emerged in the early 2000’s, the tools required to engineer living systems were under development for decades before the field of synthetic biology was officially founded and its roots can be traced all the way back to the discovery and development of genetic engineering techniques in the 1970s^3^. These scientific breakthroughs allowed scientists to manipulate DNA in unprecedented ways and opened the door for a wave of innovation in genetic engineering, such as the invention of polymerase chain reaction in 1983^4^, which allowed researchers to amplify DNA sequences for use in genetic manipulation. Not long after, in the 1990s genomics emerged as a field that involved sequencing entire genomes and provided researchers with complete maps of genomes^5^, punctuated by the completion of the human genome project in 2003^5,6^. These technological advancements have since led to the creation of a vast and ever-growing catalog of cellular components and their interactions which, in-turn, has enabled a bottom-up approach to designing regulatory networks and circuits that can be used for the biosynthesis of various biochemical products (e.g., proteins and metabolites)^7^. Moreover, the modular components within this catalog can be assembled and tested to identify combinations that yield further advantages beyond each individually. One such modular component, a genetic toggle switch, was engineered into *Escherichia coli* in the early 2000’s^8^, which resulted in the creation of the first synthetic biological system^8^. This breakthrough paved the way for further advances such as bacteria that produce biofuels or cells programmed to target cancer cells.

In the past, synthetic biologists have primarily used DNA as the molecule of choice for designing synthetic systems. As a result, synthetic DNA had been instrumental in the development of artificial genes^9^, gene regulatory networks^10^, and even entire genomes^11,12^, enabling scientists to study complex biological processes and even create synthetic organisms^2,13^. However, with the rise of RNA therapeutics, there has been a growing interest in developing synthetic systems exploiting the unique attributes of RNA molecules. RNA is more than just a messenger—it has a direct role in regulating cellular behavior and, in the past few years, large libraries of RNA parts affecting almost every step of biological control have become available^14^. RNA-based systems constructed using these libraries are safer than those constructed from DNA as they do not integrate into the host genome, making them suitable for therapeutic applications with higher safety standards^15^. Unlike DNA-based systems, RNA-based systems, built from RNA devices and circuits, are fast-acting as they do not require transcription^16^. Using diverse libraries of RNA components, researchers have begun to generate RNA-based systems that couple environmental sensing with functional outputs for therapeutic synthetic biology applications^17,18^.

The recent success of mRNA COVID-19 vaccines has sparked a surge in interest in RNA therapeutic technology and how the field of synthetic biology can be employed. Particularly, there are some challenges associated with RNA technology which can be addressed using synthetic biology. For example, base modifications and mRNA circularization have been employed to reduce mRNA immunogenicity and improve mRNA’s lifespan respectively. Each of these represents the combination of various biomolecular features in novel and synthetic ways to enhance the native function of this malleable nature of RNA. As this technology advances, scientists are discovering new opportunities for combining an RNA platform with synthetic biology in creating novel therapeutics that could revolutionize the pharmaceutical and healthcare industries. This could even include combining various types of RNA to increase its specificity and enable it to become dynamically responsive. In this review, we will explore how synthetic biology can be employed in the context of RNA technology to create powerful therapies and treatments as researchers seek to combine the two disciplines in novel ways. Specific focus will be given to how synthetic biology principles can be used to advance RNA vaccines.

### Biomedical applications of RNA in synthetic biology

**Fig. 1:**
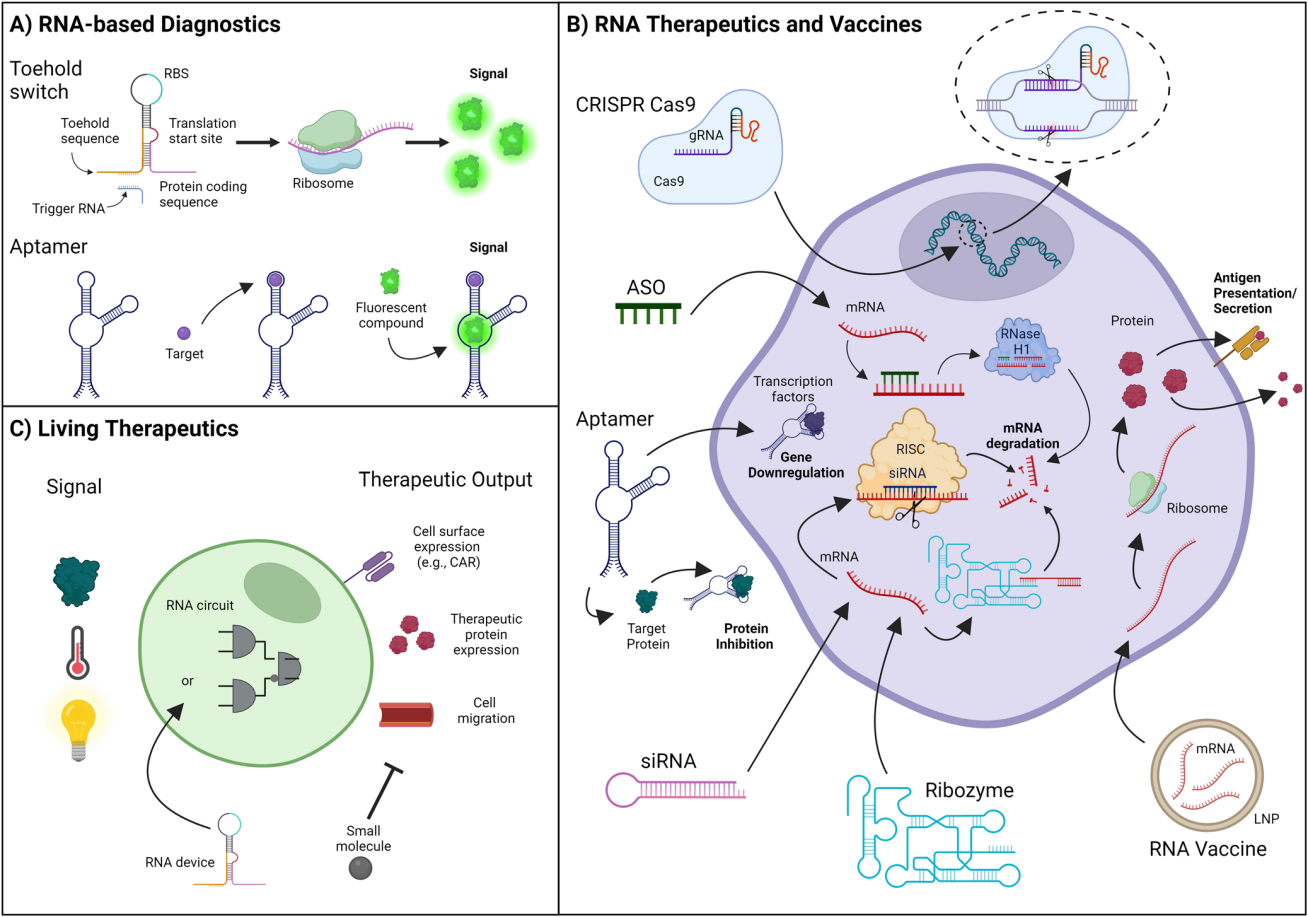
Overview of RNA-based diagnostics, therapeutics, and living therapeutics. **A** Schematic representation of RNA-based diagnostics, highlighting toehold switches detecting a trigger RNA to activate fluorescent protein expression, and aptamers recognizing a specific target molecule to generate a fluorescent signal. **B** Illustration of the cellular mechanism of action of various RNA therapeutics and vaccines, including Clustered Regularly Interspaced Short Palindromic Repeats (CRISPR) Cas9 with guide RNA, antisense oligonucleotides (ASOs), aptamers, small interfering RNAs (siRNAs), ribozymes, and mRNA vaccines. **C** Depiction of engineered RNA devices or circuits transforming cells into living factories producing therapeutic outputs, such as the expression of cell surface molecules like chimeric antigen receptors (CARs), the production of therapeutic proteins, or the initiation of cell migration. These engineered cells can also be designed to respond to small molecules as a switch to regulate therapeutic output. “Created with BioRender.com”.

RNA-based synthetic biological systems are composed of heterologous components capable of controlling gene expression in response to specific exogenous cues or endogenous metabolites. These components, referred to as RNA devices (e.g., RNA aptamers^19^, ribozymes^20,21^, and RNA switches^22^), can serve as sensors, regulators, or signal molecules. When undergoing the application of synthetic biology, these devices can either be improved through the incorporation of new parts or used in combination with other devices. When combined in a network-like structure, RNA devices can form synthetic circuits that can perform even more complex functions such as gene expression regulation^23^, signal amplification^24^, and logic operations^25^. Unsurprisingly, these devices and circuits, with their ability to control cell behavior, have already been extensively applied to the development of novel diagnostic strategies and therapeutics (Fig. 1 and Table 1)^26–29^. In addition to RNA devices, synthetic biology has also been applied to the development of RNA vaccines, including the COVID-19 mRNA vaccines developed by Moderna and Pfizer that introduced the general global population to RNA technology^30^.

**Table 1:** Technology readiness of RNA-based diagnostics, living therapeutics, RNA therapeutics and vaccines.

Technology	Mechanism	Examples	Readiness Level^a^
RNA therapeutics	mRNA degradation Genome editing Protein repression	siRNAs miRNAs CRISPR Aptamers	TLR9
RNA vaccines	Expression of antigenic protein to generate an immune response	COVID-19 vaccines	TLR9
RNA diagnostics	Structural change upon ligand binding that produces a detectable signal	Aptamer Toehold switch	TLR8
Living Therapeutics	Engineered cells that direct the immune system towards a specific target	CAR-T Cells	TLR8

*CRISPR* clustered regularly interspaced short palindromic repeats, *miRNA* MicroRNA, *siRNA* small interfering RNA, *TLR* technology readiness level.

^a^The TLR used is that defined by BARDA^137^.

#### Diagnostic tools

Early detection of disease is crucial for increasing patient survival and reducing the burden on the healthcare industry. Conventional diagnostic tools have relied on antibody-based strategies that, while highly sensitive, are costly to produce, and slow^26^. Synthetic biology, with access to a diverse catalog of RNA-based sensors and signal molecules and with its expedited development cycles, offers solutions that can overcome these limitations. In fact, a number RNA-based diagnostics have already been developed for a wide range of diseases with the potential to decrease the cost and time associated with conventional approaches with commentary on their technology readiness level listed in Table 1^31,32^.

While different RNA devices can be employed in these tools, the general strategy remains the same: design an RNA molecule that can bind to a sequence of pathogenic nucleic acid sequence, or other non-nucleic acid targets, which then results in the generation of a detectable signal (e.g., fluorescence or color change)^33^. For example, toehold switches rely on a conformation change in the structure of this RNA device following its binding to the target sequence which triggers translation of a downstream reporter gene (e.g., *gfp*). These devices have been engineered to detect a wide range of viral pathogens, including SARS-CoV-2 and other coronavirueses^34,35^, West Nile Virus^36^, Zika virus^37^, and Ebola virus^38^. In recent years, aptamer-based sensors, or aptasensors, have also gained prominence for the detection of cancer biomarkers (e.g., human epidermal growth factor receptor-2 [HER2])^39,40^, cardiovascular disease (e.g., C-reactive protein [CRP])^41,42^, neurological disease (amyloid β)^43,44^, and infectious disease^45,46^.

#### Living therapeutics

The use of RNA devices allows for the engineering of living therapeutics, such as cell therapies, that have the ability to sense and respond to environmental factors that provide information about their location, relevant disease states, and the therapeutic window timing^47^. Such cell therapies include circulating cells, implantable cells, and tissue resident human cells^47^. For example, there has been growing interest in using RNA devices to control engineered T cells (i.e., CAR T cells) to improve the safety profile and therapeutic outcomes of CAR-T therapy^17^. CAR-T cells represent a form of immunotherapy where CARs have been engineered to recognize and eliminate cells expressing tumor antigens. This approach has demonstrated profound clinical efficacy against hematological cancers^48–50^. Despite promising clinical results, severe adverse events, such as cytokine release syndrome, have complicated treatment and have occasionally proven fatal^51^. RNA devices, such as RNA switches, can be engineered to respond to cues such as changes in temperature or the presence of a small molecule to trigger the deactivation of the engineered T cells^52^. Such devices can also be designed to be activated in the presence of a specific molecule or environmental cue, triggering the expression of the CAR and enabling the T cells to target cancer cells^52^. This approach has the potential to reduce the risk of adverse events associated with CAR-T cell therapy and improve patient outcomes.

#### RNA therapeutics and vaccines

RNA devices have also become increasingly popular as a means of modulating gene expression and have enabled researchers to develop gene therapies for a range of diseases. As such, significant efforts have been made to manipulate the expression or activity of therapeutic targets utilizing three main approaches (1) ASOs, (2) siRNAs, and (3) CRISPR. Each of these strategies utilize different mechanisms to repress gene expression.

ASOs are single-stranded RNA that are complementary to mRNA and that regulate gene expression by inhibiting translation and promoting degradation of RNA^53^. ASO therapeutics can be broken down into two main categories, (1) those that cleave target mRNA and (2) those that regulate the splicing of pre-mRNA^54^. The first category of drugs cleaves target mRNA by binding to the target mRNA sequence, cleaving the sequence between the DNA and RNA duplex via RNase H activity, and thus promoting the degradation of the target mRNA^54^. FDA-approved drugs using this mechanism of action (MoA) include mipomersen and inotersen, which are used to treat homozygous familial hypercholesterolemia^55^ and hereditary transthyretin amyloidosis^56^, respectively. The second category of ASO therapeutics uses a steric hindrance-based mechanism to regulate the splicing of pre-mRNAs^54^. This MoA has been of particular interest in the treatment of inheritable diseases and the drugs golodirsen and eteplirsen, both of which target Duchenne muscular dystrophy^57^, and nusinersen, which targets spinal muscular atrophy, have already been approved^58,59^.

siRNA make use of the RNA interference (RNAi) pathway, in which siRNA interacts with Argonaute (AGO) protein to form RNA-induced silencing complexes (RICS) that suppress target mRNA expression^60^. Unlike ASOs and most other RNA pharmaceuticals, siRNA molecules are double-stranded, which facilitates their activity without chemical modification. Currently, there are only three FDA-approved drugs that take advantage of siRNA; these drugs include patisiran, givosiran, and lumasiran, which target hereditary transthyretin-mediated (hATTR) amyloidosis^61^, acute hepatic porphyria^62^, and primary hypercholesterolemia or mixed dyslipidemia^63^, respectively.

RNA aptamers also provide an effective way to control gene expression by engineering them to bind only to specific proteins that are engaged in the process of gene expression, such as transcription factors. RNA aptamers offer several advantages due to their ability to target both intracellular and extracellular molecules. Unlike other RNA-based therapeutics that require entry into the cell to perform their functions, RNA aptamers can directly bind to extracellular targets and hinder or, in some cases, stimulate their functions^32^. This method has been extensively studied and successfully employed in the treatment of age-related macular degeneration (AMD) through the use of Vascular Endothelial Growth Factor-binding Pegaptanib^64^.

The development of the CRISPR-Cas system has been revolutionary within the field of gene therapy by empowering direct and specific genome editing. This is achieved through using guide RNA (gRNA) to direct introduction of a double-stranded break in DNA^65^. To date, synthetic biology has been applied to this system in several ways. For example, RNA devices which use small molecule- and light-responsive aptamers to regulate the spacer region of a gRNA can be devised^66^. In one case, an RNA aptamer was designed to bind to an small molecule, resulting in a conformation change that prevented Cas9 from binding to target DNA^67^. Upon the removal of stimuli, however, the RNA structure of the aptamer is changed into an active state that allows a catalytically inert ‘dead’ CRISPR-Cas9 (dCas9) to bind and regulate gene expression. Other systems use riboswiches in the 5′ region of the gRNA functioning cis-acting ribozyme that exposes the spacer sequence in the presence of the aptamer ligand^68^. Furthermore, combining a CRISPR effector with an anti-CRISPR gene and microRNA (miRNA) response elements (MREs) in the 3′ untranslated region has enabled researchers to restrict gene editing to certain tissues^69^. With this method, tissue-specific MREs direct repression of the anti-CRISPR proteins and enable tissue-specific inhibition or activation of the CRISPR effector.

RNA vaccines represent an exciting new opportunity for the application of synthetic biology as they are designed to introduce synthetic RNA encoding for pathogen-specific antigens, such as the SARS-CoV-2 spike protein, into cells to trigger an immune response against target pathogens^70^. Compared to conventional vaccines, such as virus-, viral-vector-, and protein-based vaccines, the synthetic nature of RNA vaccines offers advantages in terms of speed of development, scalability, safety, and immunogenicity^71^. For example, their synthetic, cell-free production method enable mRNA vaccines can be produced quickly and at a large scale making them particularly well-suited for addressing emerging infectious diseases, such as COVID-19^72^. In fact, mRNA vaccines from Pfizer/BioNTech and Moderna were conceived of, designed, clinically tested, and granted Emergency Use Authorization in under a year^73^.

The uses of mRNA vaccines are not limited to COVID-19. Recent studies have revealed the potential of mRNA vaccines to be a powerful means of protective immunization against a variety of infectious diseases, such as influenza^74^, dengue virus^75^, herpes simplex virus type 2 (HSV-2)^76^, rabies^77^, and Zika virus^78^. There have also been efforts to develop mRNA vaccines to protect against bacterial diseases such as *Mycobacterium tuberculosis* (M.tb), one of the world’s leading infectious killers^79^. However, using host synthetic machinery to produce bacterial proteins can lead to issues such as difficulties in folding, transport, and post-translational modifications^80^. There is also currently no way to produce more complex biomolecule antigens, such as the polysaccharides found in the *Streptococcus pneumoniae* vaccines (e.g., Prevnar and Pneumovax) in human cells using mRNA vaccines, limiting their ability to compete with well-established bacterial vaccines. Additionally, mRNA vaccines are being investigated for potential use in cancer therapy^81^. Initial studies have demonstrated the ability of mRNA vaccines to induce an immune response against cancer cells, enabling the body’s own defense system to recognize and attack them, with promising results for the treatment of breast cancer^82^ and prostate cancer^83^.

### Using synthetic biology to advance RNA vaccines

**Fig. 2:**
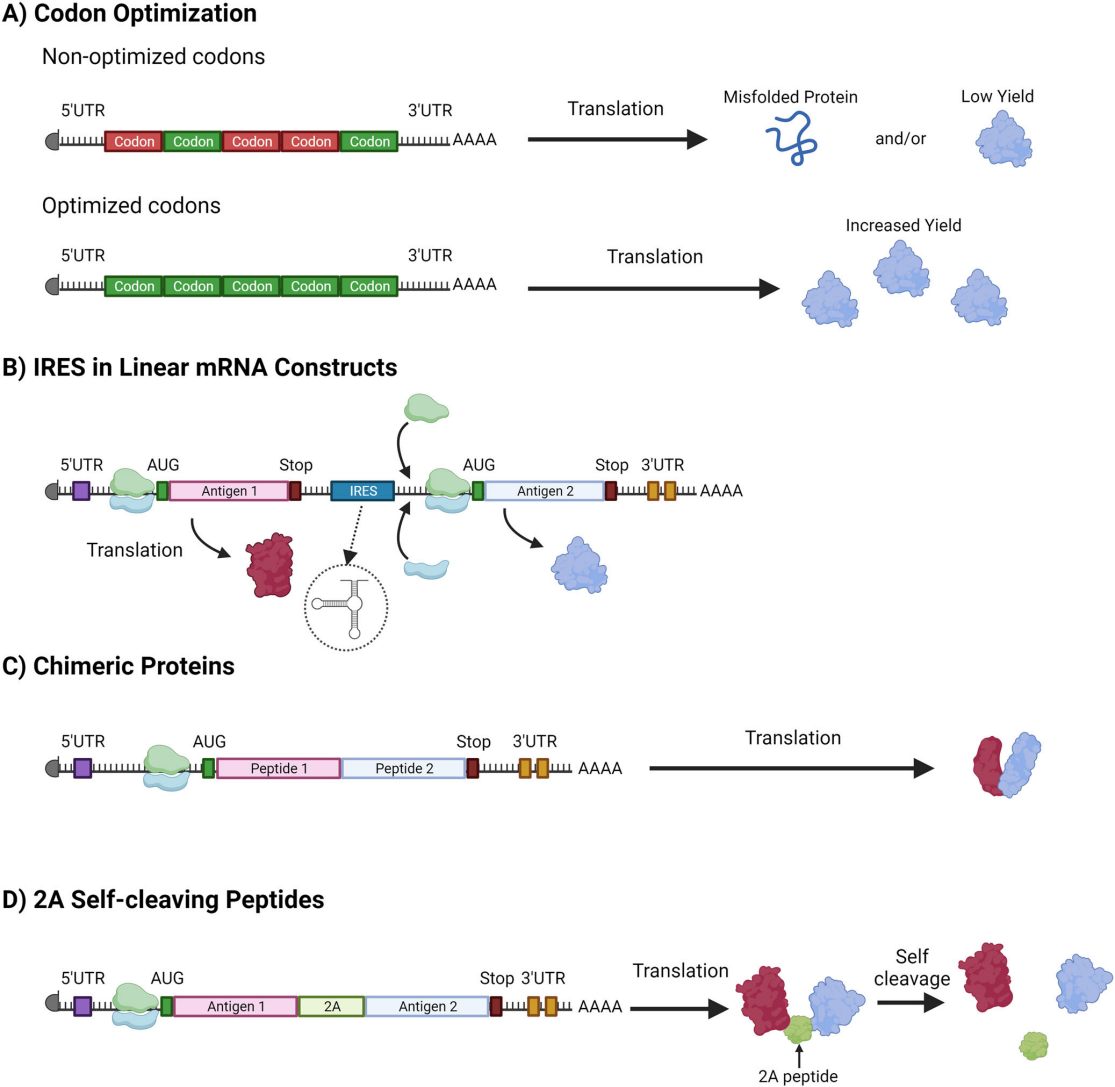
Synthetic biology tools for engineering RNA. **A** Codon optimization tackles issues with non-optimized codons that can lead to misfolded antigens or low yield when expressing antigens from non-human organisms in human cells. By substituting non-optimized codons (red) with synonymous codons optimized for human expression (green), this strategy significantly enhances the yield of correctly folded, functional antigens. **B** internal ribosome entry sites (IRES) elements function as a novel entry point for ribosomes, allowing them to bind at locations beyond the traditional 5’ Untranslated Region (UTR) on the mRNA. This feature facilitates the expression of multiple distinct antigens from a single mRNA construct. **C** The strategy for the creation of chimeric proteins fuses two distinct peptides to form a single, functional protein, enhancing the diversity of potential antigenic constructs. **D** Inclusion of 2A self-cleaving peptides serve as a molecular ‘comma’ during protein synthesis. They induce a ‘ribosome skipping’ event during translation, leading to the cleavage of the polypeptide at the 2A site. This allows for the independent expression of multiple antigens from a single open reading frame, thereby enabling multi-antigen expression from a single construct. “Created with BioRender.com”.

By taking advantage of the existing protein synthesis machinery in a transfected cell, an mRNA-based vaccine approach can turn human cells into factories for theoretically any protein antigen or therapeutic^84^. However, factors such as gene size or number, organism-dependent codons, protein confirmation, and posttranslational modifications can, in practice, limit which antigens can be effectively delivered using mRNA vaccines. However, the synthetic nature of mRNA vaccines offers many opportunities to apply synthetic biology principles to overcome such potential impediments (Fig. 2).

One such tool is codon optimization, the process by which the codons within a synthetic construct are optimized for the environment in which protein expression will occur. Each cellular environment (e.g., bacteria, viral, and eukaryotic cells) has a unique codon distribution linked with cognate tRNA molecules^85,86^. Thus, a codon bias from one cell type may result in expression challenges within another cellular environment (i.e., expressing a prokaryotic protein in a eukaryotic cell). Codon optimization can increase and improve the translation of RNA, therefore increasing antigen production. This process has been greatly advanced by the use of computational tools, such as deep learning^87^. However, there are some risks associated with codon optimization that should be considered when applying this process to vaccine design. For example, too rapid translation can result in misfolded proteins^88^. Moreover, there is evidence indicating that codon optimization is context dependent, impacted by both adjacent codons and the cellular metabolic state^89^. This suggests that further information can be gathered to enhance the application of codon optimization within pharmaceutical applications.

Due to size constraints associated with mRNA vaccines, design of vaccines for bacterial pathogens can be particularly challenging due to the need for multiple antigens to account for the complexity of the pathogen physiology or pathology. Synthetic biology principles developed for recombinant protein expression can also be exploited to overcome such impediments which will result in increased cost and complexity and may result in reduced delivery efficacy. For example, size restrictions of genetic vaccine constructs can be sidestepped by encoding for chimeric proteins, or proteins made up of immunogenic epitopes of multiple proteins^90,91^. In this way, epitopes from proteins associated with different strains, disease phases, or even different pathogens can be combined to provide more comprehensive protection. This principle is already being applied to some mRNA vaccines, including SARS-CoV-2 mRNA vaccines that encode for a chimeric spike protein^92^. Furthermore, a chimeric mRNA vaccine is under development that combines the SARS-CoV-2 spike with the influenza hemagglutinin matrix protein 1 proteins^93^.

Additional strategies to load multiple antigens onto a genetic vaccine construct include the use internal ribosome entry sites (IRESs) and self-cleaving 2A peptides. IRESs are RNA sequences used to initiate translation at an internal location within an mRNA construct, thereby facilitating the coding of polycistronic RNA. This is particularly important when designing circular RNA (circRNA) vaccines which do not have a 5’ cap to support classical cap-dependent translation^94,95^. However, there are some disadvantages to using IRESs, including size (>500 bps) and inefficient downstream gene expression that is dependent on the sequence of the gene upstream^96–99^. Self-cleaving 2A peptides represent an attractive alternative to IRESs due to their smaller size (<100 bps) and high gene expression efficiency^96,97^. These peptides, when encoded directly upstream of glycine, inhibit ribosomes from forming a peptide bond between the glycine and subsequent amino acid, thereby facilitating the expression of two separate protein antigens^100^. However, the cleavage is not 100%, which can have implications on therapeutic protein stability and activity^101^.

#### RNA vaccine constructs

**Fig. 3:**
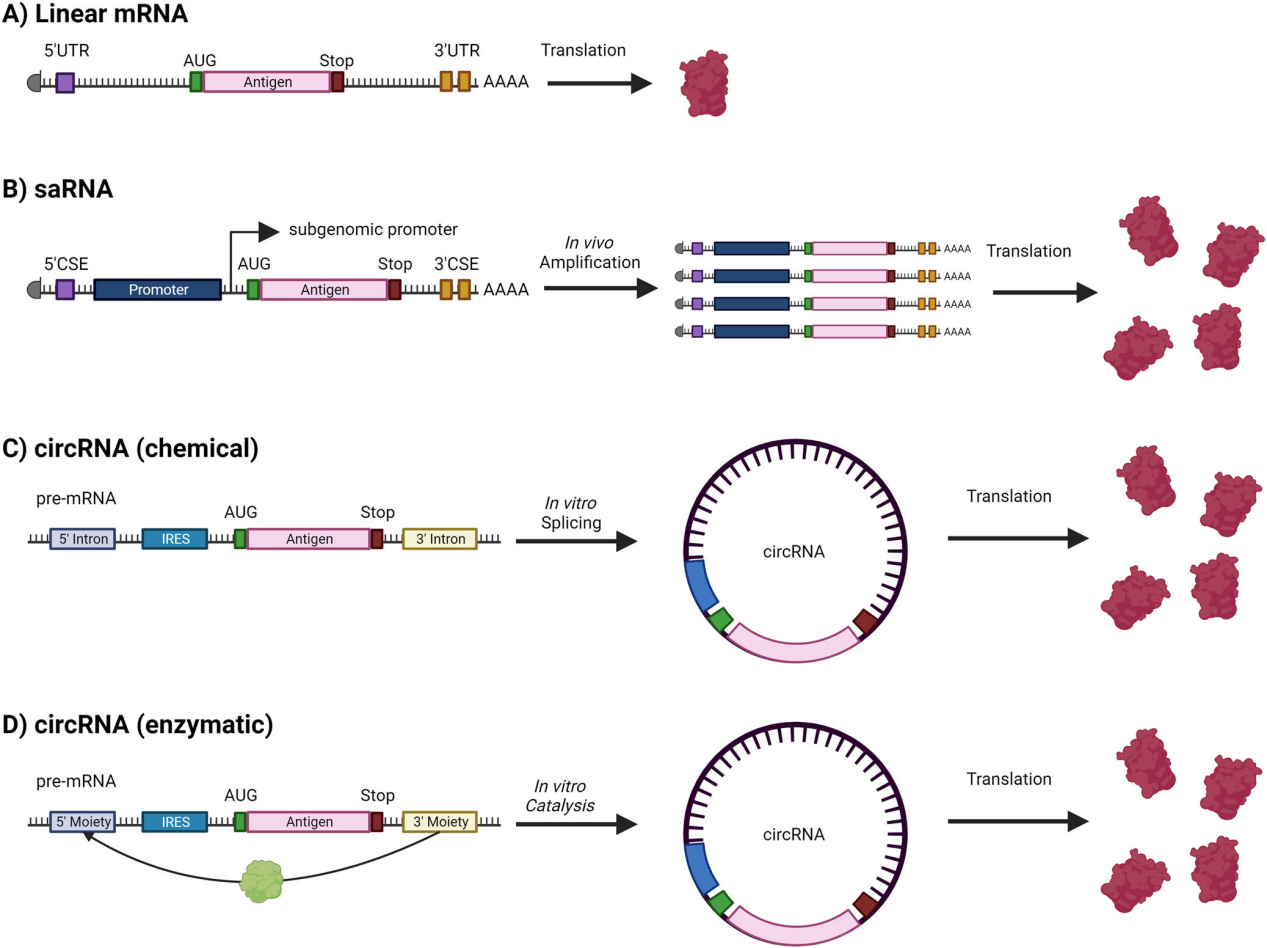
Comparative overview of linear mRNA, saRNA, and circRNA constructs. **A** For traditional mRNA vaccines, the antigenic or immunomodulatory sequence is present between 5’ and 3’ UTRs and translated directly from linear, non-replicating mRNA transcripts. **B** saRNA encodes additional replicase sequences within their construct that facilitate in vivo replication of entire mRNA construct. **C** circRNA constructs display a covalently closed loop structure created via back-splicing events that connect the 3’ end of an upstream exon to the 5’ end of a downstream exon. **D** CircRNA constructs can also be constructed enzymatically using enzymes that catalyze the formation of covalent bonds between moieties located at the 5’ and 3’ ends of the sequence. These constructs are notably stable due to their resistance to exonucleases, leading to potential prolonged antigenic protein expression within the cell. “Created with BioRender.com”.

Synthetic biology can also be applied to the RNA construct to overcome some of the existing limitations of mRNA vaccines, such as the poor stability and lifespan of mRNA within a physiological environment, which has limited success of native mRNA in pharmaceutical applications (Fig. 3). For example, modified RNA (modRNA), in which the mRNA sequence has been subjected several modifications (e.g., codon optimization, nucleic acid modification, and polyadenylation) to improve stability and expression following administration has been successfully implemented^102^. Further modification of mRNA can be done through encoding a replicase within the nucleotide sequence. This enables the mRNA to self-amplify (i.e., self-amplifying RNA (saRNA)), therefore reducing the required dose^103^. One of the more promising developments in mRNA technology has been the development of circRNA. This technology utilizes self-splicing introns to circularize mRNA into a ribonucleic acid “plasmid”. This can also be achieved through enzymatic means^104^. This provides similar improvements in stability as compared to the chemical modifications in modRNA^102^. The application of this approach has demonstrated promising results in various systems. For example, a circRNA vaccine encoding for the SARS-CoV-2 receptor-binding domain was found to induce a more potent and prolonged immune response than an equivalent dose of modRNA^105^. Importantly, the circRNA construct was also found to be highly heat stable and could be stored at room temperature for up to 2 weeks, which could facilitate easier distribution to geographical regions where a cold chain is difficult to maintain^105^. In addition to these mechanisms, origami-based approaches can be employed to generate nanoscale molecular RNA structures. Such structures have shown the ability to improve delivery and stability of such constructs^106,107^.

These advances in RNA technology potentially offer exciting opportunities to express additional factors that influence vaccine efficacy. Signal proteins, for example, can redirect the immune response of an intracellularly produced antigen, which would typically be loaded on to a major histocompatibility (MHC) class I molecule^108^. This is because signal proteins, when fused with the antigen, can lead to the excretion and subsequent loading of the antigen onto an MHC class II molecule. Additional tags, such as secretory or organelle-targeting tags can be implemented to impart delivery to specific locations as desired. Protein adjuvants can also be encoded in such a way to fuse the adjuvant with the antigen, thus leading to a more effective adaptive and innate immune response^109–112^.

The ability of saRNA and circRNA constructs to reduce the required vaccine dose while still expressing multiple antigens on the same construct may one day lead to the expression of complex processes within the host cell, such as those required to facilitate pathogen-specific posttranslational modifications or produce pathogen polysaccharides. Moreover, further application of synthetic biology to these constructs has the potential to further enhance or otherwise modulate antigen expression to address diverse applications.

#### RNA vaccine delivery

Synthetic biology principles can be applied not only to the genetic content of an RNA vaccine, but also to the vehicle in which it is delivered. The choice of delivery system for mRNA vaccines is particularly important, as its size (~1.7 MDa) can make intracellular delivery difficult^113,114^. Additionally, the negatively charged mRNA is repulsed by the negatively charged cell membrane, and is susceptible to degradation by extracellular ribonucleases^114,115^. As such, a delivery vehicle for mRNA vaccines should facilitate cellular uptake and protect mRNA from degradation^115,116^.

Characterizing biological delivery vectors, such as viruses and bacteria, allows for the use of synthetic biology tools to make targeted modifications, therefore enhancing desired delivery characteristics. One of the most notable examples of a biologic delivery vector for genetic vaccines is the adeno-associated virus (AAV), which has been used to deliver RNA encoding for the SARS-CoV-2 spike protein^117,118^. Tools have been developed to control AAV functions and have been described in detail previously^119^. In brief, these tools include synthetic biological switches (e.g., chemical, protease, or optogenetic switches) that can control cellular processes such as receptor activation, transgene expression, and protein trafficking. Gene expression following AAV delivery can also be controlled by tailoring the regulatory parts of the inverted terminal repeat-flanked expression cassette within the AAV capsid^119^. Interestingly, AAVs can also be hybridized with other viral delivery vectors, such as parvoviruses and bacteriophages, and with extracellular vesicles (i.e., exosomes) from eukaryotic cells.

Bacterial vectors, such as *Escherichia coli*, represent another potential category of delivery vehicles for genetic vaccines. A key driver of this approach is the compatibility between the molecular biology underpinnings of synthetic biology and the microbiology advantages and knowledge collected for associated microbes. *E. coli*, for example, has perhaps the most expansive knowledge base and associated molecular biology tools of any microorganism^120–122^. As a result, advanced efforts in vaccine design can be accomplished using this carrier system while taking advantage of its ability to serve as a natural adjuvant. Attenuated, nonpathogenic strains of *E. coli* represent attractive options for use in vaccine design, as many tools are available to influence antigen number, expression level, and type using this host. Moreover, other recombinant features enabled by synthetic biology tools could serve in ways to assist with antigen delivery. For example, certain endosomal lysis proteins such as listeriolysin, natively associated with the intracellular pathogenic bacterium *Listeria monocytogenes*, facilitate the endosomal escape and cytosolic trafficking required to better deliver antigenic content to antigen presentation cells during administration of an engineered bacterial-based vaccine carrier^123–127^.

One drawback to antigen delivery within the confines of a microbial delivery vehicle is its biological complexity. Though bacterial or viral particles offers potential advantages with regards to adjuvant patterning and synthetic biology tools, there are other biological features that do not enhance vaccine efficacy. These same features may have a negative impact on the overall vaccine design, as extraneous elements of the microbial vaccine carrier system may spur unwanted immune reactions or cause undesirable toxicity. Furthermore, bacteria are prone to the degradation of mRNA and are thus not suited for its delivery. However, by using synthetic biology tools (e.g., DNA synthesis), it is possible to construct a synthetic, minimalized bacterial genome within a hollowed-out cellular framework^11,128,129^. Such a synthetic cell would be designed for a specific biological purpose with minimal extraneous cellular features.

Genetic payloads can also be delivered in fully chemical carrier systems, such as liposomes. Liposomal delivery systems have often accompanied studies in gene and drug delivery^130,131^, but the approach gained wider recognition due to their use in the COVID-19 mRNA vaccines^132,133^. Such liposomes represent an intersection of antigen delivery with synthetic biology as they can be further modified to impart delivery specificity. For example, there have been multiple strategies developed for the attachment of proteins to liposomal surfaces^134–136^. These mechanisms can be adapted to surface localization of an antibody or similar targeting ligand to directly deliver RNA to a specific tissue. This would enable directed RNA therapy in an approach like radioimmunotherapy.

### Outlook

The union of synthetic biology and RNA technology has led to remarkable advances in medical treatments, allowing us to create novel therapeutics and vaccines that hold the potential to revolutionize global health care. However, the application of this technology is still in its early stages and has not come close to reaching its full potential.

We are currently in the midst of rapidly advancing artificial intelligence (AI) and machine learning that will further expand the horizons of RNA therapeutics and vaccines. AI and advanced computer-based models can be used to suggest modifications to RNA sequences for enhanced efficacy, identify novel disease targets, and computationally design specialized RNA sequences, such as aptamers and encoded antibodies, with precise specificity to unique targets. For example, we can imagine the use of AI to analyze large datasets of genetic information and proposing RNA construct designs for the expression of complex, multidomain proteins such as monoclonal antibodies. Antibody sequence and binding information can be utilized to train AI to propose antibody sequences with desired target or neutralizing abilities. We can also envision the use of AI to identify novel regulatory and ribozyme functions that could be incorporated into RNA medical products in order to detect specific disease states such as an increase in blood glucose levels in diabetes patients. Such regulatory and ribozyme functions could be used to develop an “on/off switch” that would then stimulate the production of insulin, thereby providing precise blood sugar level control for patients.

The application of AI and machine learning to synthetic biology can also potentially be used to overcome current limitations of mRNA vaccines, such as an inability to express multidomain antigens or polysaccharide antigens. Without the capability to produce these antigens, RNA vaccines will remain limited in the diseases it can target and will not be able to fully replace traditional vaccines. AI could potentially help design complex circuits that could carry out the multistage processes required for production of such antigens and that could be administered as an RNA vaccine, thereby opening new possibilities for vaccine development.

While there are still many challenges to be tackled when it comes to using synthetic biology for developing vaccines and RNA therapeutics, the potential is undeniable. With continued research and innovation, we may soon be able to see groundbreaking new technologies that can improve the lives of millions. Through careful consideration and creative design, synthetic biology can provide an effective and safe platform to develop novel treatments for a range of diseases. In the years ahead, we are sure to see amazing advances in this field that could change the face of healthcare forever.

### Reporting summary

Further information on research design is available in the Nature Research Reporting Summary linked to this article.

### Supplementary information

Reporting Summary
